# Immediate full-arch fixed rehabilitation of a narrow mandible with newly conceived connection system implants: A case report

**DOI:** 10.34172/japid.2024.010

**Published:** 2024-04-28

**Authors:** Gerardo Pellegrino, Zoran Zaccheroni, Giuseppe Lizio

**Affiliations:** ^1^Department of Biomedical and Neuromotor Sciences (DIBINEM), University of Bologna, Bologna, Italy; ^2^Private Practice, ZEA Dental Clinic, Imola (BO), Italy; ^3^Private Practice, Messina (ME), Italy

**Keywords:** Alveolar bone loss, Case report, Dental implant, Dental prosthesis retention, Immediate dental implant loading, Metal-on-metal joint prostheses

## Abstract

Rehabilitating thin jaws without reconstructive surgery entails using narrow implants. The proposed treatment adopted an innovative implant system, allowing the mini-screws to be parallel and immediately loaded. A mandible, wearing an overdenture, was functionalized contextually to the residual dental extraction and the placement of six 2.4-mm thick one-piece implants. Low-profile intermediate abutments, the LEMs, able to rotate over the spherical heads of the fixtures, were connected after suturing, oriented, and blocked in a mutual parallel position. The copings, engaging with a tapered juncture of the LEMs, resulted in their alignment to be intraorally wedged together. The provisional superstructure enclosed the copings and was immediately connected to the implants, and the definitive prosthesis was delivered after three months. No clinical signs of peri-implantitis or radiographically evident bone loss were recorded after a two-year follow-up without any prosthetic complication. No cases have been published regarding mini-implants bearing fixed prosthesis rehabilitation.

## Introduction

 In cases of thin alveolar bone, narrow/mini implants with < 3.3 mm diameter are limited to support missing incisors’ single crowns or removable overdentures, particularly in the preliminary phases of treatments.^1-3^ These one-piece monophasic thin fixtures cannot house an internal screw connection for the fracture risk and cannot bear fixed prostheses. The most frequently described indications were the edentulous arch and single arch non‐load‐bearing teeth in the anterior region.^4,5^ The reported mean survival rate was 94.7 ± 5% (range: 80%–100%) with a mean follow‐up of 34 ± 20 months (range: 12-78 months).^4^ Only one study indicated an implant success rate of 92.9% with a mean marginal bone loss ranging from 0.6 to 1.43 mm.^5^ A further problem is the implant divergence since the position of the fixtures must depend on the residual bone, with difficulties in obtaining complete passivation of the prosthesis.^6^ A screwed retention system cannot fasten the snap-joint connection of a mini-implant after the engagement with intermediate flat abutments. The misalignment, even minimal, cannot be corrected. Consequently, the horizontal atrophies, particularly in the molar zones, are intended to be resolved onlywith a pre-implantology surgical reconstruction procedure.^7^

 Provided adequate implant primary stability, the immediate loading protocols in full-arch rehabilitations were as reliable as the two-stage ones, preventing patients from wearing a transitional removable denture and a second surgical approach for fixture retrieval,^6^ even in atrophic situations.^8-11^ Mini-implants allow an immediate temporary connection with a resinous denture to reduce the trauma to the hard and soft tissues before the final fixed prosthetic structure.

 The adaptation and connection of the provisional screw-retained prosthesis in the same surgical session are further complicated by the intraoral wounds, especially in an open-flap approach. Indeed, the retrieval of the screw housing in the fixtures’ heads after the implant-abutment coupling and the insertion of the connection screw through the occlusal access of the abutment is not simple at all, particularly in the case of intermediate flat abutments necessary for implant divergence.^6,12-13^ As alternatives, a cemented connection, easier to be passively fitted, has the shortcoming of the cement entrapment in peri-implant tissues.^14,15^ At the same time, the conical frictional joint tolerates only minimal implant dis-parallelism.^16,17^ The snap-fit approach can simplify the connection procedure, and similar systems have been proposed in the literature.^12,14,18^

**Figure 1:**
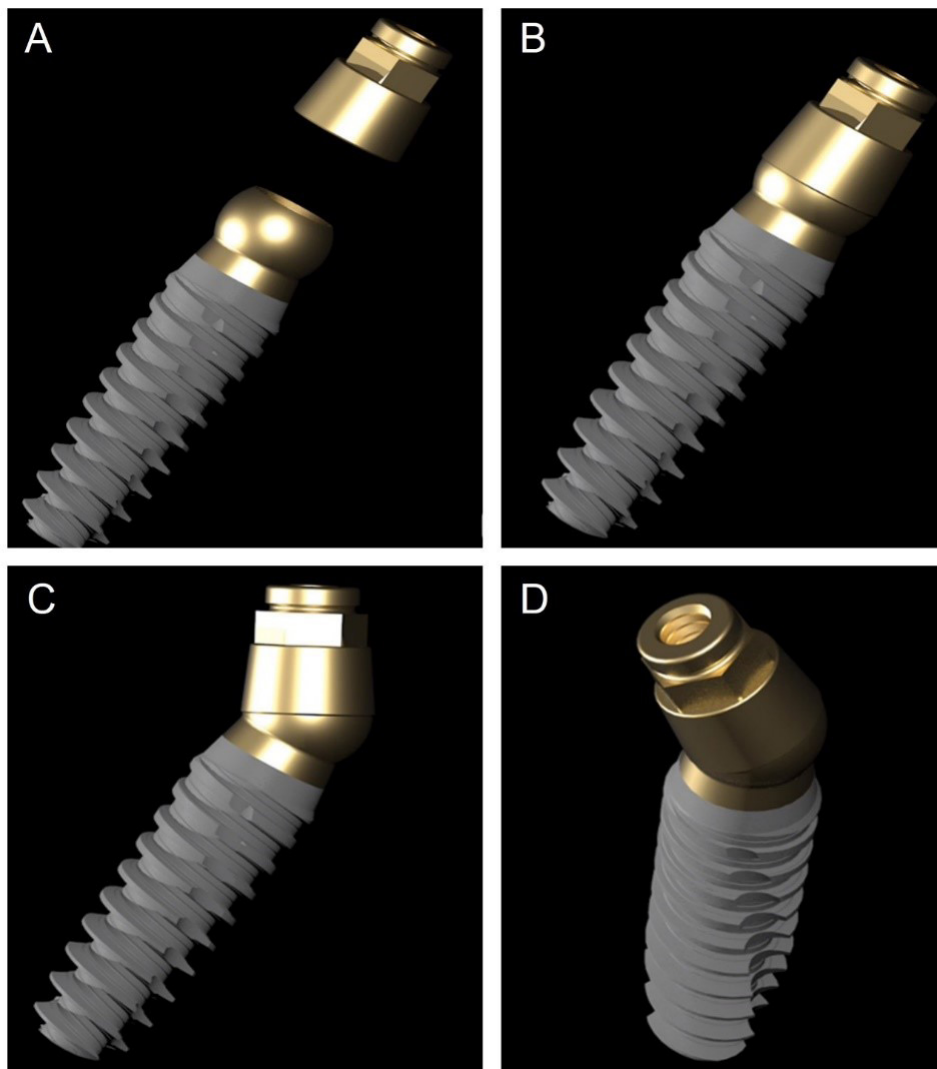
The one-piece fixture aligned with the “LEM” before connection (A). The “LEM” connected to the fixture with the same axial spatial direction (B). The maximal “LEM” rotation relative to the fixture axis around the spherical but-joint (C). A different projection of the complex fixture-LEM

 The Orbit implant (Bionica®, Thiene (VI), Italy) consists of a one-piece fixture with a spheric butt-joint to be snap-fit to a cylindric intermediate flat-abutment called “LEM,” embracing it to be rotated up to 30° relative to the implant long axis. After finding the correct position, the abutment is blocked by an external ferrule screw, correcting the misalignment. The pillar to be enclosed in the prosthetic framework can be connected with a screw-retained or conic frictional modality (Figure 1). Due to the absence of an internal screw housing, the fixture of the Orbit system presents a solid structure, particularly of its head portion, the same for all implant diameters, ranging from 2.4 to 6.0 mm.

 This paper reports a knife-edge atrophic mandible immediately rehabilitated with a full-arch screw-retained prosthesis, proposing mini-implants as an alternative to long-lasting surgical treatment to rehabilitate horizontally atrophied jaws.

 The novelty of the present case consists of the one-session surgically fixed prosthetic treatment of a very thin mandible thanks to the particular connection design of the here-adopted mini-implant system. To the best of our knowledge, this is the only system that associates the advantage of the one-piece narrow implants with the possibility of immediate loading with an absolute passivation of prosthetic coupling. The particular snap-joint connection obtains intraoperative parallelism of the pillars, correcting their spatial orientation and fixating it thanks to the external ferrule, which can be rapidly closed and opened.

## Case Report

**Figure 2:**
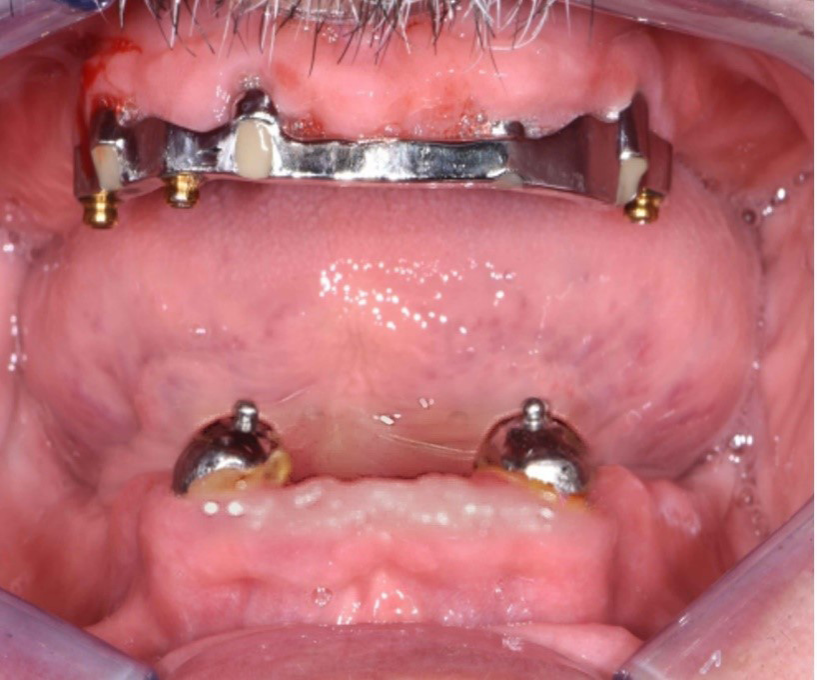
Starting clinical situation after the removal of the superstructure

**Figure 3:**
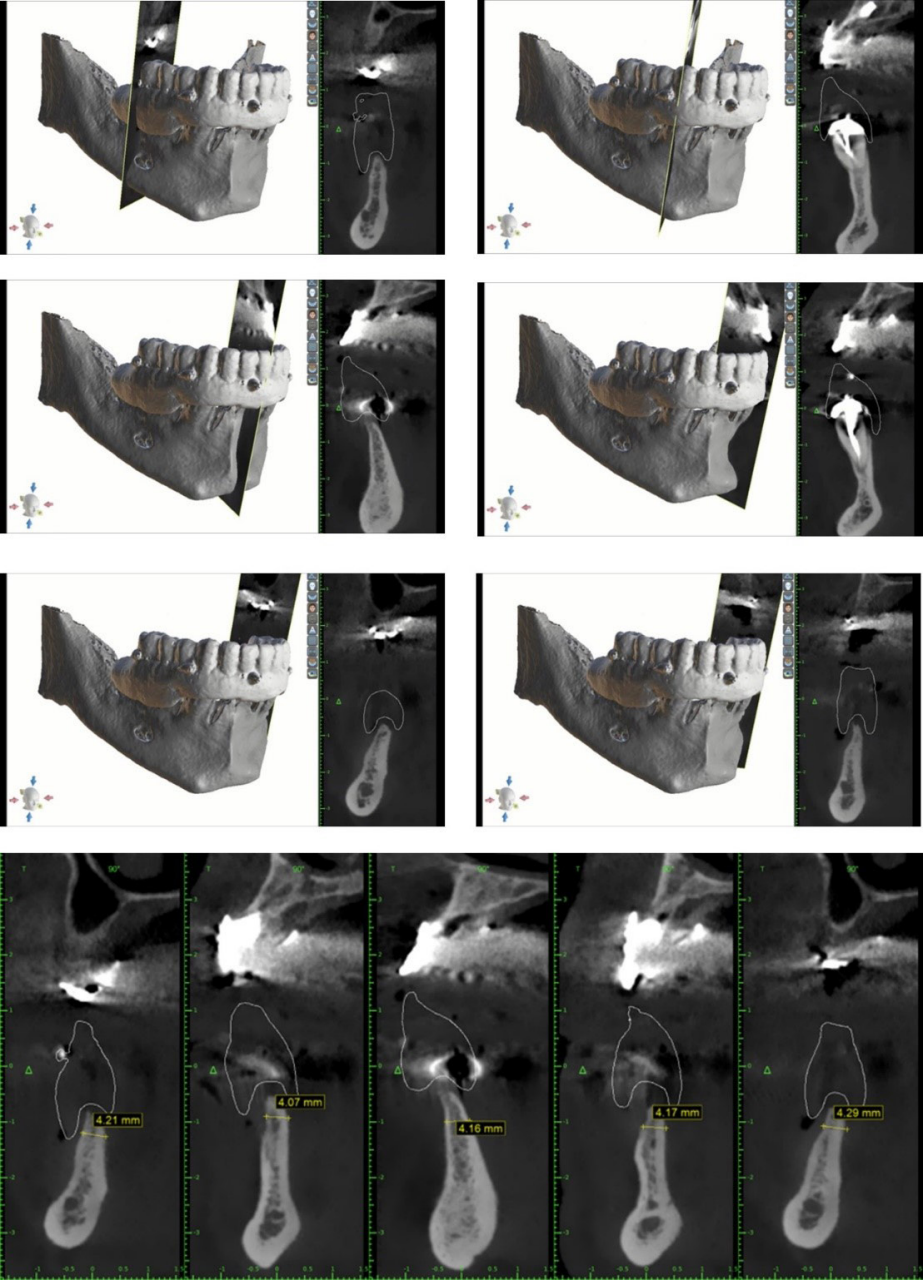
CBCT slices after matching the bone data with the STL files from denture scanning. The adopted imaging software was 3D Romexis, Planmeka, Fin- 00880 Helsinki, Finland.

 The present case report follows the CARE checklist (https://www.care-statement.org/checklist). A 60-year-old partially edentulous male patient, complaining of functional and aesthetic discomfort, visited our dental clinic. The patient wore an implant-supported bar-engaged removable denture in the upper jaw. At the same time, the mandible was rehabilitated with an overdenture retained by two ball attachments on the residual cuspid roots (Figure 2). A clinical intraoral examination showed an improvable occlusal relationship between the arches, with poor lower denture stability. The patient requested to be treated as quickly as possible and was willing to wear a fixed mandibular prosthesis immediately. After taking a panoramic x-ray, the occlusion was balanced, and a correct vertical dimension was achieved between the jaws by adjusting the overdentures. The inferior prosthesis was scanned, and the standard tessellation (STL) data were imported to the dedicated software. A cone-beam CT of the jaws was taken, and the relative DICOM files were digitally acquired and matched with the STL ones. The residual mandibular alveolar process appeared dramatically reduced in the horizontal dimension, with about a 5-mm thickness in the frontal and posterior zones (Figure 3). The option of the mini-implant was proposed to the patient since he firmly kept his decision to undergo one-session treatment despite being clearly warned about the unknown predictability of such an approach.

**Figure 4:**
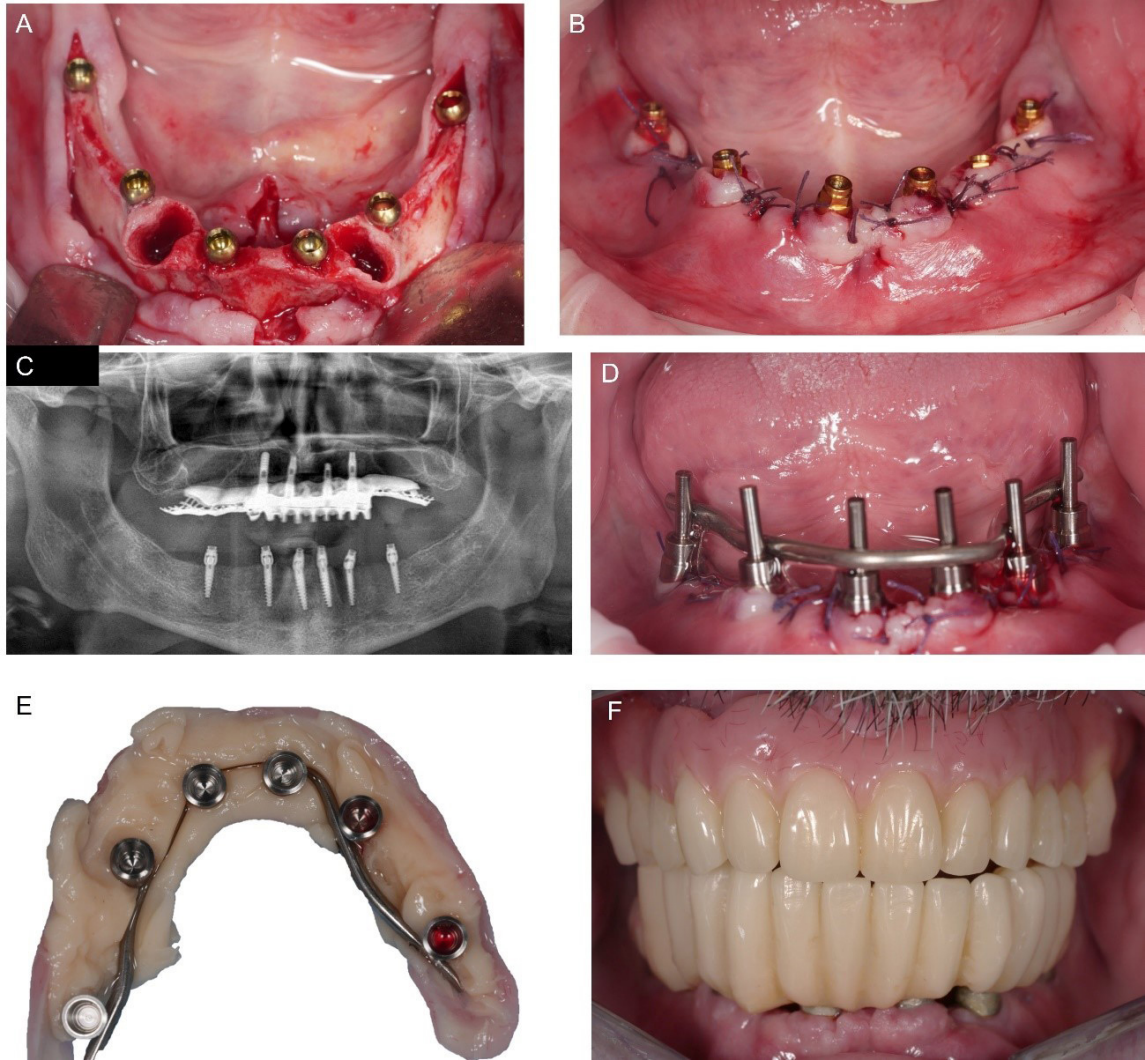
Intraoperative phases. Situation after the implant placement (A) and “LEM” connection (B). Panoramic x-ray to verify the correct implant position (C). Intraoral welding after the copings were matched (D). Mucosal surface of the provisional denture enclosing the copings (E) and intraoral placement (F)

 After a standard antibiotic prophylactic regimen (2 g of amoxicillin one hour before the procedure), a full-thickness flap with vestibular and lingual anterior releasing incisions was raised from the bone under local anesthesia. The cuspid dental roots were extracted, and six implants measuring 2.4 mm in diameter, Orbit (Bionica, Thiene (VI), Italy), were placed in 36/37, 34, 31/32, 41/42, 44, 46/47 correspondent tooth locations, all with the same length, 13 mm, apart from the rear ones, 11,5 mm (Figure 4A). The intermediate flat abutments, the LEMs, were snap-fit-connected to the fixtures’ heads, orientated parallel to each other, and blocked in the decided position turning the external ferrule screw, and the flap was sutured. (Figure 4B & 4C). The straight copings were connected with a frictional external Morse tapered joint to the intermediate abutments, intraorally welded together (Figure 4D), and enclosed in the provisional prosthetic structure, which was relined, refined (Figure 4E), and connected to the implants (Figure 4F).

 Ibuprofen (800 mg, three times a day for the following three days, and then, as needed) was prescribed along with a soft diet and chlorhexidine mouthwashes.

**Figure 5:**
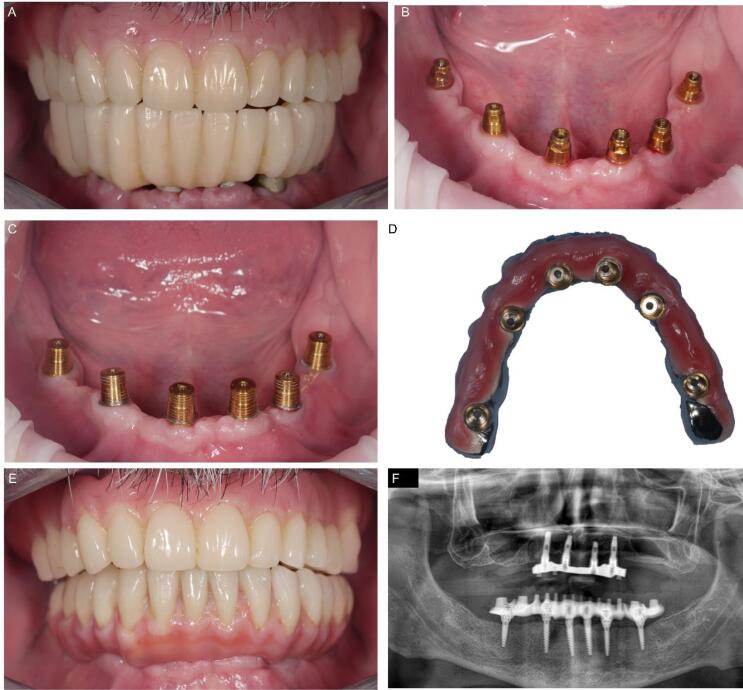
Final prosthesis delivery. Clinical situation three months after treatment with (A) and without (B) the temporary superstructure. Cap positioning and activation (C), and cementation of these inside the definitive prosthesis (D). Final placement of the definitive device (E), and panoramic x-ray after one year(F)

 The patient reported minimal hematoma and ecchymosis in the submandibular region, which resolved within ten postoperative days. The control checks were carried out weekly in the first month after the immediate loading and every 15 days until the definitive prosthesis connection session. No complications or problems were recorded. After three months, the definitive structure was connected with a conical joint system. In the meantime, a new removable denture for the upper jaw was projected and connected to the metallic framework to correct the occlusal relationship with the new mandibular rehabilitation. No complications or radiological peri-implant bone resorption occurred at a weekly follow-up rate in the first month and monthly in the first two years after surgery (Figure 5).

 The procedures reported are under the ethical standards of the responsible committee on human experimentation (institutional and national) and with the Helsinki Declaration of 1975, as revised in 2008.

## Discussion

 The anatomic situation associated with tooth extraction implicated an axial misalignment of the fixtures due to the necessity to engage adequately consistent bone. MUA or University of California of Los Angeles (UCLA) devices, as intermediate angled flat-abutment systems connected to the fixtures to compensate for their misalignment, rely on the retaining screws, which, particularly under the no-axial loadings, are subjected to preloading force loss and fracture.^9,19^ The enlargement of the screw entails a bulkier profile limiting the space for the superstructure and soft tissue management.

 The first advantage of the implant system adopted in the present case report was the availability of fixtures with a 2.4-mm diameter in the head portion right under the transgingival sphere, tapering to 2.0 mm in the apex. Mini-implants, measuring ≤ 2.5 mm in diameter, have been used with a mean survival rate of 94.5 ± 5% (range: 80%–100%) after a 12–78-month observation period. The number and the quality of studies on this topic are scarce.^20^ Mini-implants consist of one-piece devices with an external extra-osseous snap-joint to reduce the fracture risk, remaining high in the collar portion. Such a design does not allow optimal soft tissue management and a qualitative connection with the supra-structure. Hence, the mini-implant indications are reduced in retaining complete dentures, particularly in the mandible, in replacing mandibular incisors and maxillary lateral incisors, and in temporary supporting fixed restorations, especially for single anterior tooth replacement.^1,20,21^ Flanagan and Mascolo indicated using 1.8-3.3-mm thick implants to support fixed full-arch rehabilitation, up to ≥ 11.5 mm in height, with ≥ 8 fixtures for the mandible and ≥ 10 for the maxilla. In fixed prosthetics, rounded flat cusps, splinting, implant protective occlusal schemes, and placement only in dense bone sites are considered mandatory for successful mini-implant treatment.^22^ No cases of fixed rehabilitation on these devices have been reported in the literature until now.^5^

 The concept of a ball-shaped attachment fastened by an external ferrule screw being part of the intermediate abutment, like in the Orbit system, allowed realizing a one-piece full-metal fixture maintaining the same 4.0-mm diameter of the sphere, despite the limited thickness of the fixtures’ body. Therefore, the mini-implants were able to support occlusal loadings with less risk of fractures of the joint, favoring the immediate connection with the prosthesis.

 The Orbit spherical interlock allows up to 30° of inclination in a 360° excursion range without needing anti-rotational mechanisms whose presence would condition the abutment axial orientation. Hence, the same components can be used regardless of fixtures’ dimensions and orientation, without the need for internal connecting screws, simplifying the immediate prosthesis connection and adaptation.

 After resolving the misalignment of the mini-implants fixing and blocking the LEMs in the correct axes, the copings, connected to the intermediate abutments with a Morse taper juncture, could be more easily welded together, achieving complete passivation of the superstructure.^23,24^ Degidi et al,^25^ after a 6-year follow-up period, reported a cumulative mean marginal bone loss of 1.39 mm (SD = 0.67) for the implants placed in the maxilla (n = 124) and 1.29 mm (SD = 0.71) for the implants placed in the mandible (n = 87), demonstrating the intraoral welding technique’s success in rehabilitating fully edentulous patients with a fixed and immediate prosthesis.

 Intraorally welding further reduced the risk of mini-implant overloading, preserving the osseointegration and allowing the intraoperative immediate loading.^26^

 Immediate loading with a provisional fixed prosthesis allows the dentist to reline the superstructure and condition the soft tissue, avoiding the interim use of a removable denture. The occlusal function can be checked from the beginning without peri-implant bone loss, favoring the final prosthesis realization and delivery. The literature about immediate loading protocols entails few controlled studies, with significant heterogeneity of clinical situations and limited follow-ups.^27,28^ The survival rates of immediately loaded implants range from 87.5% to 100% at 1 to 5 years.^29^ Focusing on edentulous jaws, implants loaded contemporarily during the surgical session, compared with conventional loading as the control group, exhibited a 98.3% survival rate after eight years of follow-up in the maxilla^28^ and 98% after one year in the mandible.^24^ Pera et al^30^ achieved the same results with an immediate loading protocol of 4-6 post-extraction implants as a conventional loading of fixtures placed in healed sites in the upper jaw. Controlled occlusal loads for full-arch fixed rehabilitation are supposed to favor implant osseointegration differently from a single unit and partial prosthesis.^27^

 The external Morse taper connection between the LEMs and the pillars was chosen for the definitive rehabilitation, simplifying the removal and insertion of the prosthesis in control sessions.

 A one-piece implant used in this case report can help remove the abutment–fixture gap from the bone, reducing the risk of micro-movements at the interface and consequent bacterial contamination.^31^ A marginal bone loss of 1.5 to 2 mm below the connection around two-piece dental implants in the first loading year is reported in the literature as a routine finding even related to the reentry for the soft tissue healing abutments engagement.^32^ Moreover, the one-piece fixtures facilitated the flap suturing, maintaining most ball-head exposures.

 The “sphere-acetabulum” like joint realizes a sealed connection distant from the bone ridge without interfering with the mucosa healing around the implant neck. In this regard, the Morse taper frictional implant‒abutment joint obtained better outcomes in terms of peri-implant bone resorption, realizing a deeper internal coupling between the fixture and the abutment without the need for a screw or cementum and behaving as a single-piece device.^33,34^ The tapered fit allowed a thinner abutment concerning the fixture platform and the consequent “platform-switching” related bone overgrowth over the joint and adequate soft tissue height maintenance.^32^ Nevertheless, the tapered junction is incompatible with the implants’ dis-parallelism, and angled abutments are required to obtain a superstructure passive connection. The intraoral welding technique obtains a passivity of the prosthesis joining together, but even in this approach, tilted intermediate components were also necessary to obtain a correct emergence profile.^35^

 The Orbit system was more straightforward than the other ones. Only one-shape straight abutment, different only in the mucosal portion height, is necessary, independently from the shape and fixture inclination, within the 30° range.

 The snap-joint and the external fastening ferrule make the prosthetic delivery easier without the use of small tightening screws, avoiding retrievability problems of implant slots and engagement.

 Similar snap-retention attachments have been conceived to simplify the procedure. Two “micro-locking” systems are based on the elasticity and shape memory of the retentive portion made of nickel-titanium (Ni-Ti) alloys, which, under physical stimuli, change their status and position, enabling the insertion and disinsertion of the prosthetic superstructure.^12^ In particular, one system consisted of metallic flaps and the inner and outer of the abutment surface, which, under an electromagnetic induction effect, acquires an opened or closed umbrella-like position to engage or release the fixtures and the prosthetic crown.^13^ A second attachment involves zirconia (ZrO_2_) made of spherical sub-components fixed to a metallic spring inside the pillar. These spheres, framed in the undercut of the low-profile joint, realize effective retention without needing a screw. The pillar/abutment, cemented inside the prosthetic crown, can be easily removed manually. A clinical retrospective study with up to 2 years of follow-up reported 100% implant survival with minimal peri-implant bone loss and no prosthetic complications using this type of connection.^12^

 Another conceived joint entails a semi-spherical intermediate abutment to be matched with an interchangeable plastic spring at the coupling portion of the pillar. The last cited connection tolerated high dis-parallelism of the fixtures and seemed helpful in fixed prosthetic full-arch rehabilitation of atrophic jaws without bone reconstructive procedure.^36,37^ Two studies verifying the reliability of the last cited attachment, similar to the Orbit system, revealed no variations in stress distribution in a virtual mandibular all-on-four rehabilitation with and without the fastening screws in the anterior implants.^38,39^ The computational finite elements analyses (FEM) were fundamental in understanding the properties of these attachments before their clinical use, limited up to now to a two-year follow-up,^17^ and can support the realization of future studies in vitro and in vivo, now missing, to evaluate the Orbit attachment performance better. The similarity with hip articulation could take advantage of studies about hip prostheses in the literature.^40^

 The metallic implant materials used in orthopedic and traumatic surgery, cobalt-chrome alloy or titanium, despite being more resistant than ceramic materials, revealed the shortcoming of osteolysis related to wear-induced particles,^40,41^ particularly in metal-on-metal bearing couple implants in joint prostheses.^42^ Metal-free materials, i.e., high-performance polymers, were proposed, and the most popular became polyether-ether-ketone (PEEK). A reinforced version of PEEK has a similar elasticity as the human cortical bone, can be sterilized, and avoids scattering phenomena under diagnostic irradiation.^41^ As an alternative, coatings with bioceramics or treatments conditioning the microstructure of the superficial layers were considered. Jamari et al reported that surface texturing of the hip implants with a dimple bottom geometry reduced pressure and wear of the prosthesis contact areas.^42^ Following the orthopedic research, dental implants’ titanium surfaces were physically and chemically conditioned and covered to improve their bioactivity. Bioceramic coatings with hydroxyapatite, with different percentages of carbonate, improved the interaction of the fixtures with the bone cells and growth factors, enhancing the osseointegration.^43^ PEEK can be used in implant dentistry for its mechanical and physical properties, similar to bone. However, the surface should be improved for better interaction with the bone environment.^44,45^

 Under functional loading, the wear and corrosion of the fixture and abutment coupling surfaces can affect the implants’ success. A one-piece implant such as Orbit places the fixture‒abutment gap above the marginal bone level, reducing the risk of wear and related osteolysis. The caps connected to the LEMs are covered with titanium nitride to contrast wear and tear better. In vitro and in vivo studies should focus on this issue, too.^41^

 The use of 2.4-mm fixtures for the rehabilitation of both edentulous jaws was reported by Worni et al.^42^

## Conclusion

 Thanks to its newly conceived connection, the proposed implant system allowed the loading of the mini-screws during the surgical session, obtaining a passive prosthesis fitting. The prosthetic Morse tapered connection with straight coping abutments and the possibility of welding together the pillars intraorally could be a new opportunity for rehabilitative simplification protocols.

## Acknowledgments

 The authors thank the Orbit implant (Bionica®, Thiene (VI), Italy) for furnishing graphic materials to support clinical images.

## Competing Interests

 The authors declare no conflicts of interest.

## Data Availability Statement

 Not applicable.

## Funding

 The authors declare no external funding.

## Informed Consent Statement

 Written informed consent was obtained from the patient to publish this paper.
